# Radiocarbon Production Events and their Potential Relationship with the Schwabe Cycle

**DOI:** 10.1038/s41598-019-53296-x

**Published:** 2019-11-19

**Authors:** A. Scifo, M. Kuitems, A. Neocleous, B. J. S. Pope, D. Miles, E. Jansma, P. Doeve, A. M. Smith, F. Miyake, M. W. Dee

**Affiliations:** 1 University of Groningen, Centre for Isotope Research, Nijenborgh 6, 9747AG Groningen, The Netherlands; 2 University of Cyprus, Department of Computer Science, 1 University Avenue, 2109 Aglantzia, Cyprus; 3 NASA Sagan Fellow, Center for Cosmology and Particle Physics and Center for Data Science, New York, NY USA; 4 Oxford University, Oxford Dendrochronology Laboratory, Mill Farm, Mapledurham, Oxfordshire, RG4 7TX United Kingdom; 5 Cultural Heritage Agency of The Netherlands, Smallepad 5, 3811 MG Amersfoort, The Netherlands; 6 Australian Nuclear Science and Technology Organisation (ANSTO), New Illawarra Rd, Lucas Heights, NSW 2234 Australia; 7 Nagoya University, Institute for Space-Earth Environmental Research, Chikusa-ku, Nagoya, 464-8601 Japan

**Keywords:** Atmospheric chemistry, Solar physics

## Abstract

Extreme cosmic radiation events occurred in the years 774/5 and 993/4 CE, as revealed by anomalies in the concentration of radiocarbon in known-age tree-rings. Most hypotheses point towards intense solar storms as the cause for these events, although little direct experimental support for this claim has thus far come to light. In this study, we perform very high-precision accelerator mass spectrometry (AMS) measurements on dendrochronological tree-rings spanning the years of the events of interest, as well as the Carrington Event of 1859 CE, which is recognized as an extreme solar storm even though it did not generate an anomalous radiocarbon signature. Our data, comprising 169 new and previously published measurements, appear to delineate the modulation of radiocarbon production due to the Schwabe (11-year) solar cycle. Moreover, they suggest that all three events occurred around the maximum of the solar cycle, adding experimental support for a common solar origin.

## Introduction

Since the discovery of annual anomalies in the radiocarbon record, also known as Miyake Events, much effort has been undertaken to determine their cause^1–4^. Detected through the analysis of tree-ring archives, the events comprise sudden and globally synchronous increases in the atmospheric concentration of radiocarbon ($\Delta$^14^C). The first two verified anomalies occurred in the years 775 and 994 CE (henceforth Event-775 and Event-994). The uplifts were quickly attributed to increases in cosmic radiation, as radiocarbon is produced by means of nuclear reactions associated with the cosmic ray cascade^5,6^. The rate of production is proportional to the cosmic ray flux entering the Earth’s magnetosphere, which is in turn modulated by the interplanetary magnetic field in accordance with the Schwabe cycle. In fact, this solar cycle is inversely related to radiocarbon production as, during periods of maximal solar activity, the shielding of cosmic rays by the solar plasma in the interplanetary and the geomagnetic fields is enhanced. In such cases, a decrease in radiocarbon production occurs, and vice versa^7–9^. This sinusoidal variation has already been measured in tree-ring samples by various groups, and exhibits a peak-to-trough amplitude of around 4–6‰^8,10–12^. Despite this anticorrelation, the most widely accepted theory for the cause of the anomalous increases in $\Delta$^14^C is extreme solar storms^3,13^. This is largely because of the occurrence frequency of the events, as several have now been attested and others predicted during the Holocene^4,14,15^, but it is also the conclusion of modelling studies of isotopic signatures in natural archives^16^. However, to date few direct connections have been made between the Miyake Events and established solar behavior, or instrumentally monitored solar storms. Indeed, some analyses still contradict the claim of a solar origin altogether^17,18^. Here, our results suggest that Event-775 and Event-994 both occurred during the period of the 11-year solar cycle when the Sun was most active. This provides additional support for the hypothesis that these anomalies were instigated by powerful solar emissions.

The most intense solar storm to hit Earth in modern times was the Carrington Event of September 1859 CE. In this case, the solar flare itself was directly witnessed^19^, and the magnetic excursions recorded at Kew Observatory in London outstripped measurement capacity^20^. The so-called magnetic crochet associated with the impact of the initial soft x-ray/extreme ultra-violet flux on the ionosphere remains one of the largest ever reported for the mid-latitudes; and intensified aurorae, instigated by the geomagnetic disturbance, were observed as far south as the tropics^20^. Further consequences, such as malfunctions to telegraph systems, were also recorded world-wide^21^. Despite these dramatic impacts, the Carrington Event left no detectable imprint on the atmospheric radiocarbon record^22^. However, continuous and reliable observations of the solar cycle were already being made at the time of the Carrington Event. This was achieved by recording the number of sunspots (known as the International Sunspot Number, ISN). Hence, it was quickly established that the storm took place six months prior to the maximum activity of the solar cycle.

Historical solar activity may be elucidated by carrying out very high precision AMS measurements on series of annual tree-rings spanning the events in question. In our study, Event-775 and Event-994 are analyzed at levels of precision that allow us to claim evidence of the modulation of atmospheric radiocarbon due to the 11-year Schwabe cycle^11,23,24^. As a result, we are able to assign a possible timing for these events in relation to the solar cycle, an outcome that has several important implications. Firstly, if each event occurred around the maximum of the solar cycle, like the Carrington Event, this would provide additional support for a common solar origin. To elaborate, solar flares and coronal mass ejections (CMEs) are in general more frequent and more intense during times of highest solar activity. For example, the recorded ISN and the number of higher energy flares (M-class and X-class flares) are very well correlated (r = 0.95, r^2^ = 0.90); although, significant flares can occur throughout the whole period^25^. Secondly, establishing whether the Carrington Event, Event-775 and Event-994 all occurred at the same point on the Schwabe cycle may suggest further shared characteristics, such as whether the mechanisms that drove Event-775 and Event-994 can be regarded as extreme versions of those that initiated Carrington.

## Results

In this study, we make use of 169 radiocarbon data; of these, 76 are new measurements and 93 come from previously published datasets. The suite of new results, averaged per year, is given in Table 1. The Supplementary Information (SI) contains the full set of newly obtained data (Table S1), the previously published datasets (Table S2) and details about how duplicate measurements were dealt with, including the outputs of chi-squared statistical comparisons (Table S3). The new results were obtained at an average precision of 1.71‰ (1 σ) per year, with reported uncertainties encompassing counting statistics, normalization and sample preparation calculated in accordance with standard data reduction procedures^26^.

**Table 1:** Results of the radiocarbon analysis over the Carrington Event, Event-775 and Event-994.

Sample A (Oak) – Carrington			Sample B1 (Juniper) – Event-775		
Calendar Age (year CE)	^14^C age (yr BP)	$\Delta$^14^C (‰)	Calendar Age (year CE)	^14^C age (yr BP)	$\Delta$^14^C (‰)
1853*	126 ± 11	−3.94 ± 1.40	756	1350 ± 15	−23.35 ± 1.82
1855	137 ± 16	−5.55 ± 2.01	758	1344 ± 15	−22.86 ± 1.82
1856*	149 ± 11	−7.03 ± 1.36	759	1360 ± 25	−24.92 ± 3.03
1857	119 ± 13	−3.56 ± 1.55	762–763	1305 ± 16	−18.63 ± 1.98
1858	111 ± 13	−2.69 ± 1.55	765	1316 ± 16	−20.27 ± 1.98
1859*	144 ± 8	−6.89 ± 0.99	766	1311 ± 15	−19.78 ± 1.83
1860*	130 ± 10	−5.03 ± 1.24	768	1324 ± 16	−21.61 ± 1.97
1861	145 ± 13	−7.26 ± 1.54	769	1308 ± 15	−19.77 ± 1.83
1862*	133 ± 9	−5.89 ± 1.08	771	1326 ± 24	−22.20 ± 2.89
1863	158 ± 13	−9.10 ± 1.54	772	1320 ± 18	−21.59 ± 2.13
1864*	140 ± 11	−7.00 ± 1.40	775	1172 ± 16	−3.76 ± 2.01
1865*	137 ± 9	−6.75 ± 1.08	777*	1177 ± 11	−4.62 ± 1.39
1866	119 ± 13	−4.64 ± 1.54			
**Sample B2** (**Oak**) **– Event-775**			**Sample C** (**Juniper**) **– Event-994**		
**Calendar Age** (**year CE**)	^**14**^**C age** (**yr BP**)	$\Delta$^**14**^**C** (**‰**)	**Calendar Age** (**year CE**)	^**14**^**C age** (**yr BP**)	$\Delta$^**14**^**C** (**‰**)
770*	1290 ± 7	−17.72 ± 0.86	976	1136 ± 15	−23.32 ± 1.82
771*	1278 ± 12	−16.32 ± 1.45	978	1108 ± 15	−20.15 ± 1.83
772*	1281 ± 6	−16.87 ± 0.76	980	1113 ± 18	−20.99 ± 2.13
773*	1308 ± 11	−20.20 ± 1.39	982	1143 ± 16	−24.88 ± 1.97
774	1285 ± 25	−17.56 ± 3.06	984	1120 ± 16	−22.32 ± 1.98
775*	1211 ± 15	−8.63 ± 1.85	986*	1102 ± 11	−20.36 ± 1.37
776*	1142 ± 15	−0.19 ± 1.87	988	1116 ± 18	−22.31 ± 2.13
777*	1153 ± 14	−1.65 ± 1.69	989–991*	1096 ± 12	−20.11 ± 1.52
778*	1168 ± 10	−3.66 ± 1.25	993	1047 ± 16	−14.47 ± 1.99
779*	1176 ± 14	−4.78 ± 1.69	995	1035 ± 16	−13.23 ± 1.99
			997	1045 ± 16	−14.70 ± 1.99

*Years where pretreatments were repeated and duplicate measurements obtained and averaged (See S1, S2 and S3, SI).

Investigation of radiocarbon modulation due to the solar cycle has previously been conducted in different ways. Burchuladze *et al*.^27^ directly compared the ISN with radiocarbon measurements on vintage wine samples^27^; Stuiver & Braziunas^10^ applied a cubic spline interpolation to annual tree-ring data and evaluated the residuals between the subsequent fit and a moving average^10^; and Güttler *et al*.^11^ used a band-pass filter to analyze the signal in order to extract the hidden periodicities from tree-ring data^11^. Of these three approaches, the first one can only be applied over the time since the ISN has been recorded, therefore we make use of it only in the case of the Carrington Event. Of the other two, we concentrate on the band-pass filter, but we have also completed some analyzes using the residuals from the spline interpolation, which can be found in S7 (SI). In relation to the filter, we used a Butterworth band-pass filter^28^ which was designed to extract periodicities between 8 and 20 years (see Güttler *et al*.^11^). It is important to note that applying a digital filter to a dataset will undesirably but unavoidably introduce an offset, as the output of the filter will be altered from the input signal^29^. This happens because using a filter with fixed cut-off frequencies and order will shift sinusoids of different frequencies by different amounts. In our case, the filter will introduce a variable offset of 3 years or less.

### Comparing Δ^14^C with ISN over the Carrington Event

**Figure 1:**
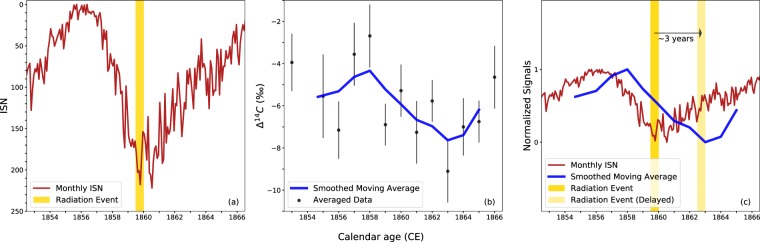
Left, monthly ISN (red, see S5 (SI) for further details) and timing of the radiation event (yellow). Center, averaged results of the 21 measurements on Sample A (1-sigma error bars). Smoothed moving average (blue) obtained using a 3-year moving average smoothed by a Savitzky-Golay filter^44^. Right, normalized monthly ISN (red) and smoothed moving average (blue). The delay between the two signals (~3 years) is an approximation of the radiocarbon atmospheric residence time.

For this study, we made use of 21 radiocarbon measurements over 13 single-year tree-rings. Multiple measurements were performed on 7 of the samples, and the results were averaged (see Table S1a, SI). Due to the high precision of the radiocarbon measurements obtained, a broad modulation of the Δ^14^C data can be observed, with a peak-to-trough amplitude of about 5‰. This value is consistent with the effect of solar modulation pointed out in previous analyzes of the Schwabe cycle^8,11,30–32^. Moreover, when presented together with the ISN data (see Fig. 1), it is clear that the ^14^C data follow the same pattern (the ISN axis is inverted to account for the inverse relationship between the two parameters). As also expected, the two signals were not perfectly aligned, as the sunspot record reflects immediate solar behavior, while the radiocarbon record is delayed due to the so-called residence time of the isotope in the atmosphere. By measuring the cross-correlation between the normalized $\Delta$^14^C and the normalized ISN data, one can estimate the delay time between the two signals. Here this residence time is estimated to be 3 ± 1 years, which is in good agreement with previous estimates^33,34^. As can be seen, our Δ^14^C data approximately follow the sinusoidal profile of the solar cycle, and hence mimic the variability of incoming solar radiation.

### Event-775

For this study, we made use of a total of 58 radiocarbon measurements over 32 single-year tree-rings. Of these, 15 are new measurements from sample B1, 27 are from sample B2, and 16 were previously produced by Miyake *et al*.^2^. Multiple measurements were performed on 11 of the 32 single-year tree-rings, and the results were averaged for each year (see Tables S1b and S1c, SI).

**Figure 2:**
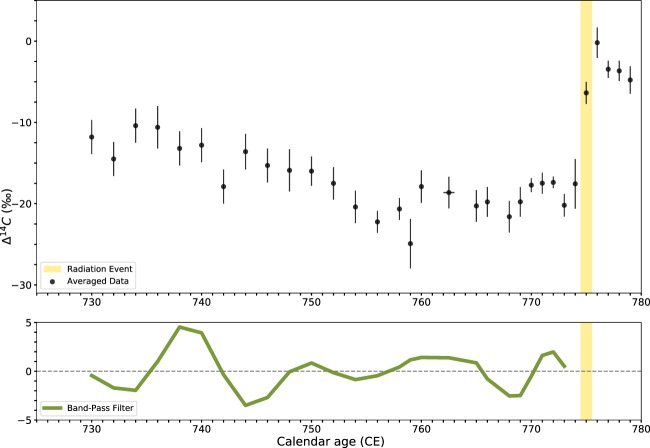
Results of the analysis over the Event-775. In the top row, averaged data points with 1-sigma error bars (black) and timing of the radiation event (yellow). In the bottom row, the result of a Butterworth band-pass filter applied to the $\Delta$^14^C data (green). The band-pass filter is only applied to the data prior to the spike, in order to not affect the periodicity.

The radiocarbon results are in close agreement with previous measurements over the event^1,3^. The outcome of our analysis of the data is shown in Fig. 2. From the averaged Δ^14^C data leading up to the spike in the year 775 CE, a sinusoidal pattern is clearly evident. This is especially true for the decades immediately preceding the event, where we achieve the highest precisions. We observe approximately four cycles within this interval of 45 years, which have a peak-to-trough amplitude that is similar to the Carrington Event (~5‰), and a period of approximately 11 years in length. This pattern was accentuated by the residuals from the cubic interpolation (see S7, SI). Thus, we note that Event-775, like the Carrington Event, appears to occur when solar activity is at its maximum (Fig. 2).

### Event-994

For this study, we made use of a total of 90 radiocarbon measurements over 48 single-year tree-rings, although on this occasion, due to availability of tree rings, the radiation event is located towards the middle of the series. Of these, 13 are new measurements from sample C, including 2 replicated measurements (see Table S1d, SI). This data was complemented by 13 results from Damon *et al*.^35^; 7 from Dee *et al*.^36^; 25 from Menjo *et al*.^37^; and 32 from Miyake *et al*.^23,35–37^.

**Figure 3:**
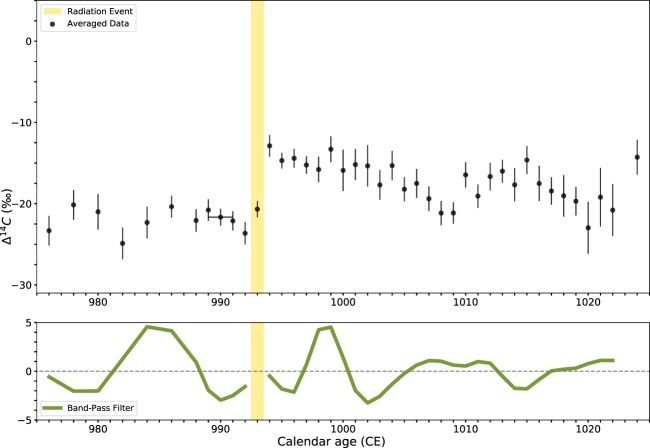
Results of the analysis over the Event-994. In the top row, averaged data points with 1-sigma error bars (black) and timing of the radiation event (yellow). In the bottom row, the result of a Butterworth band-pass filter applied to the Δ^14^C data (green). The band-pass filter is applied separately before and after the spike.

Once more, the radiocarbon results of the newly measured data are in good agreement with previously published results^2^. Individual tree-rings prior to the Event-994 spike were sampled every other year, apart from the last data point (989–991 CE), which came from three tree-rings too narrow to separate. Our band-pass filter analysis of the data is shown in Fig. 3, while the spline residuals are presented in S7 (SI). From the averaged Δ^14^C data leading up to and after the spike, a sinusoidal pattern is again evident. They indicate that approximately 4.5 cycles occurred within this interval of 50 years. It should be noted that the band-pass filter was applied separately before and after the spike, in order to limit the disruption caused by the sudden increase in $\Delta$^14^C. However, for at least a decade afterwards the output is still clearly influenced by the radiocarbon spike (Fig. 3).

It is also important to point out that this peak occurs in 993 CE, one year before the recognized date for Event-994, but this is consistent with several other studies (see Büntgen *et al*.^38^). In this case, we also tentatively interpret the patterns in our data as evidence of the solar modulation of atmospheric radiocarbon production. Therefore, we note that Event-994 also appears to occur when solar activity is at its maximum (Fig. 3)^38^.

### Monte Carlo resampling

**Figure 4:**
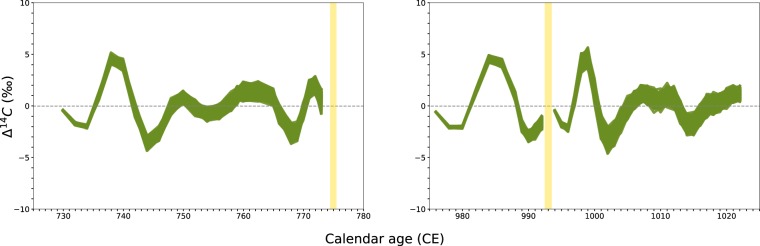
Butterworth band-pass filter applied to Monte Carlo resampling |(1,000 times) of the normally distributed experimental data analyzed in Figs. 2 and 3. The yellow vertical band represents the year in which the event occurred, which was at least one year before the peak in Δ^14^C, for Event-775 and Event-994. For both events, the intersection between the yellow vertical band and the data interpolation corresponds to maximum activity of the sun within the Schwabe cycle or lowest radiocarbon production rate.

In order to establish if the results of our numerical analyzes were influenced by a possible low signal-to-noise ratio in our datasets, we performed Monte Carlo simulations in which we randomly resample our datasets 1,000 times, assuming the data was Normally distributed. Then, we applied the same Butterworth band-pass filter over the resampled datasets. In Fig. 4, we report the outcome of these analyzes for both the events in terms of $\Delta$^14^C versus sample growth year (CE). For Event-775 and Event-994, the results are in accordance with the outputs shown in Figs. 2 and 3, albeit more distinct and robust. Hence, we conclude that the Carrington Event, Event-775 and Event-994 all appear to have occurred near the point of maximum activity of the solar cycle.

## Discussion

Our results present a coherent picture across all three solar events. We observed a moderate variation in radiocarbon over the Carrington Event of 1859 CE; our new and previously published data over Event-775 followed a sinusoidal pattern with peak-to-trough amplitude of about 5‰, and a periodicity of about 12 years; likewise, our Event-994 data varied with a 5‰ amplitude and period of around 11 years. Although it cannot be stated categorically, we believe that the most parsimonious explanation for the undulating pattern in all our datasets is the solar modulation of radiocarbon production. No other repeating process of this same magnitude and duration is easily conceivable. This finding is best exemplified by the Monte Carlo resampling, as it accounts for the inherent variability in estimates of radiocarbon concentration.

In all three cases, the radiation events occur in close proximity to the point of maximum activity of the 11-year solar cycle. Although it did not leave any radiocarbon signature, the Carrington Event of 1859 CE was already known to have occurred around the point of maximum activity of the solar cycle, due to contemporary accounts of the sunspot record. Direct comparison between our new Δ^14^C data over the Carrington Event and such sunspot counts also allowed us to make an estimation of 3 ± 1 years for the residence time of radiocarbon in the atmosphere at this time. In relation to Event-775 and Event-994, our data also show that the radiation events occurred when the sun was at its most active, and radiocarbon production exhibited a local minimum.

In summary, our finding strengthens the likelihood of a solar origin for Event-775 and Event-994, and provides valuable experimental evidence of a link between them and the Carrington Event.

## Methods

**Figure 5:**
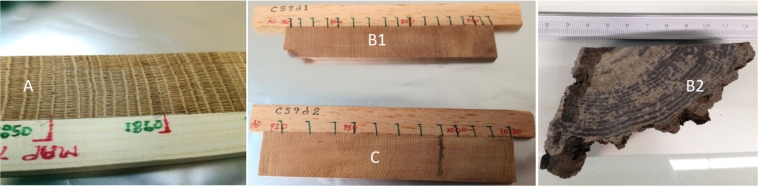
Left, Sample A, the oak piece analyzed for the Carrington event. As is evident in the photo, the wood exhibits marked and distinct annual growth rings. Center, Samples (**B**1) (above) and (**C**) (below), juniper wood analyzed for the study of Event-775 and Event-994. The growth rings are once more evident but finer and at times more warped than in Sample (**A**). Right, Sample (**B**2), oak wood analyzed for Event-775. Growth rings are marked and distinct over most of the sample.

Four different dendrochronologically dated wood samples were obtained and pretreated, each containing distinct annual growth rings spanning the years of the three different events. Sample A, covering the Carrington Event, was a piece of oak from southern England (see Fig. 5). Samples B1 and C, spanning Event-775 and Event-994, respectively, were two pieces of juniper, which grew in the Sierra Nevada Mountains, California, at an altitude of about 3000 m. These samples were obtained from the Oxford Dendrochronology Laboratory, England. A further oak sample which also traversed Event-775, B2, was obtained from the Cultural Heritage Agency of The Netherlands. The rings corresponding to the years of interest in sample B1 were quite thin, while the ones from Sample C were somewhat irregular in shape, which made separation challenging. Nonetheless, the possibility of material from adjacent rings being mixed together was considered minimal.

The photosynthetic uptake of ^14^CO_2_ is indistinguishable from its stable isotopic analogues, save a degree of readily correctable mass-dependent fractionation. Much of the carbon absorbed is immediately locked into the cellulosic structure of the growth-rings, and chemical exchange between rings is negligible^39,40^. Hence, the optimal extract for reconstructing past $\Delta$^14^C levels is the fraction known as alpha-cellulose. The method used for such extractions at Groningen is described in detail by Dee *et al*.^41^, so only a brief summary is presented here. The wood samples first undergo a physical preparation procedure, including elimination of extraneous soil and particulates from the bulk material, cutting each growth ring from the main sample, and then slicing or crushing each into small fragments. The sample is then chemically pretreated with an intense Acid-Base-Acid procedure, namely HCl (5.47% w/vol (1.5 M), 80 **°**C, 20 min); NaOH (17.5% w/vol, 60 min, RT) with ultra-sonication under an N_2_ atmosphere; HCl (5.47% w/vol (1.5 M), 80 **°**C, 20 min), followed by a strong acidified oxidant (NaClO_2_, 1.5% w/vol in HCl (0.06 M), 80 **°**C, 20 hrs), in order to extract the alpha-cellulose fraction. Each step is separated by a thorough rinse to neutrality with deionized and decarbonized water. Next, the alpha-cellulose fraction is combusted, and the CO_2_ liberated cryogenically trapped, reduced to graphite and pressed into Al cathodes^42,43^. The radiocarbon content of graphite extracted from each sample is then determined by AMS (200 kV MICADAS, Ionplus). In order to achieve very high precision measurements, the samples are measured for longer periods than usual.

## Supplementary information

Supplementary Info

Dataset 1

## Data Availability

All data generated or analyzed during this study are included in this published article (and its Supplementary Information files).
