# Supplementary material for: Radiocarbon Production Events and their Potential Relationship with the Schwabe Cycle
Source: Sci Rep. 2019 Nov 19;9:17056. doi: 10.1038/s41598-019-53296-x (PMC6863917; doi:10.1038/s41598-019-53296-x)
Supplement: Supplementary file 1 — Supplementary Info [file 41598_2019_53296_MOESM1_ESM.pdf]

Radiocarbon Production Events and their Potential Relationship with the Schwabe Cycle - *SUPPLEMENTARY INFORMATION*

Scifo A., Kuitems M., Neocleous A., Pope B.J.S., Miles D., Jansma E., Doeve P., Smith A. M., Miyake F. & Dee M. W.

S1 – RAW DATA (NEWLY MEASURED)

Sample type	*Sample number (GrM)*	*Calendar age (year CE)*	*^{14}C age (year BP)*	*$\Delta^{14}C$ (‰)*	*Averaged ^{14}C age (year BP)*	*Averaged $\Delta^{14}C$ (‰)*
A (Oak) Mapledurham (UK)	13331	1853	124 ± 15	-3.70 ± 1.86	126 ± 11	-3.94 ± 1.36
	13404		129 ± 16	-4.32 ± 2.01		
	13332	1855	137 ± 16	-5.54 ± 2.01	137 ± 16	-5.54 ± 1.98
	13334	1856	169 ± 16	-9.62 ± 2.00	149 ± 11	-7.03 ± 1.36
	16797		131 ± 15	-4.92 ± 1.86		
	12714	1857	119 ± 13	-3.56 ± 1.55	119 ± 12	-3.56 ± 1.49
	12715	1858	111 ± 13	-2.68 ± 1.55	111 ± 12	-2.68 ± 1.49
	12716	1859	142 ± 13	-6.64 ± 1.55	144 ± 8	-6.89 ± 0.99
	16798		153 ± 14	-8.00 ± 1.70		
	18107		138 ± 13	-6.15 ± 1.85		
	12717	1860	114 ± 13	-3.30 ± 1.55	130 ± 10	-5.03 ± 1.24
	13335		153 ± 16	-8.12 ± 2.00		
	12719	1861	145 ± 13	-7.25 ± 1.54	145 ± 12	-7.25 ± 1.48
	12721	1862	127 ± 11	-5.15 ± 1.39	132 ± 8	-5.89 ± 1.11
	12727		140 ± 13	-6.76 ± 1.55		
	12722	1863	158 ± 13	-9.10 ± 1.54	158 ± 12	-9.10 ± 1.48
	13336	1864	146 ± 15	-7.74 ± 1.85	140 ± 11	-7.00 ± 1.36
	13405		134 ± 16	-6.26 ± 2.01		
	12725	1865	139 ± 11	-6.99 ± 1.39	137 ± 8	-6.75 ± 1.11

	12729		135 ± 13	-6.50 ± 1.55		
	12726	1866	119 ± 13	-4.64 ± 1.55	119 ± 12	-4.64 ± 1.48

Sample type	***Sample number (GrM)***	***Calendar age (year CE)***	***^{14}C age (year BP)***	***Δ^{14}C (‰)***	***Averaged ^{14}C age (year BP)***	***Averaged Δ^{14}C (‰)***
B1 (Juniper)	16801	756	1350 ± 15	-23.35 ± 1.82	1350 ± 15	-23.35 ± 1.82
	16803	758	1344 ± 15	-22.86 ± 1.82	1344 ± 15	-22.86 ± 1.82
	16804	759	1360 ± 25	-24.92 ± 3.03	1360 ± 25	-24.92 ± 3.03
	16805	762-763	1305 ± 16	-18.63 ± 1.95	1305 ± 16	-18.63 ± 1.95
	16806	765	1316 ± 16	-20.27 ± 1.95	1316 ± 16	-20.27 ± 1.95
Sierra Nevada (USA)	16809	766	1311 ± 15	-19.78 ± 1.83	1311 ± 15	-19.78 ± 1.83
	16810	768	1324 ± 16	-21.61 ± 1.95	1324 ± 16	-21.61 ± 1.95
	16811	769	1308 ± 15	-19.77 ± 1.83	1308 ± 15	-19.77 ± 1.83
	16812	771	1326 ± 24	-22.20 ± 2.92	1326 ± 24	-22.20 ± 2.92
	16813	772	1320 ± 18	-21.59 ± 2.19	1320 ± 18	-21.59 ± 2.19
	16814	774	1261 ± 16	-14.62 ± 1.96	1291 ± 12	-18.29 ± 1.47
	17197		1333 ± 19	-23.41 ± 2.31		
	16815	775	1172 ± 16	-3.76 ± 1.98	1172 ± 16	-3.76 ± 1.98
	16816	777	1193 ± 16	-6.60 ± 1.98	1177 ± 11	-4.62 ± 1.36
	17198		1161 ± 16	-2.64 ± 1.98		

Sample type	***Sample number (GrM)***	***Calendar age (year CE)***	***^{14}C age (year BP)***	***Δ^{14}C (‰)***	***Averaged ^{14}C age (year BP)***	***Averaged Δ^{14}C (‰)***
B2 (Oak)	11849	770	1314 ± 17.5	-20.62 ± 2.13	1290 ± 7	-17.72 ± 0.86
	12731		1286 ± 14	-17.2 ± 1.68		
	12786		1276 ± 16	-15.98 ± 1.99		
	14094		1290 ± 11	-17.69 ± 1.38		
Dorestad (NL)	10567	771	1312 ± 22.5	-20.5 ± 2.74	1278 ± 12	-16.32 ± 1.45
	11774		1267 ± 15	-15 ± 1.84		
	12686		1250 ± 37.5	-12.91 ± 4.61		
	10568	772	1295 ± 22.5	-18.54 ± 2.75	1281 ± 6	-16.87 ± 0.76
	12179		1286 ± 14	-17.44 ± 1.68		
	12737		1266 ± 14	-14.99 ± 1.69		
	12739		1271 ± 14	-15.61 ± 1.68		
	14093		1292 ± 11	-18.18 ± 1.38		
	10569	773	1315 ± 22.5	-21.1 ± 2.74	1308 ± 11	-20.20 ± 1.39
	13095		1312 ± 19	-20.73 ± 2.29		
	13096		1298 ± 19	-19.03 ± 2.29		
	10570	774	1285 ± 25	-17.56 ± 3.06	1285 ± 25	-17.56 ± 3.06
	10572	775	1205 ± 25	-7.84 ± 3.09	1211 ± 15	-8.63 ± 1.85
	17405		1215 ± 19	-9.08 ± 2.31		
	10574	776	1125 ± 25	1.97 ± 3.12	1142 ± 15	-0.19 ± 1.87
	13098		1152 ± 19	-1.4 ± 2.33		
	10575	777	1165 ± 25	-3.13 ± 3.1	1153 ± 14	-1.65 ± 1.69

	14839		1148 ± 16	-1.02 ± 2.02		
	10577	778	1170 ± 25	-3.87 ± 3.1	1168 ± 10	-3.66 ± 1.25
	14440		1173 ± 15	-4.25 ± 1.86		
	15240		1162 ± 16	-2.88 ± 2.02		
	10578	779	1170 ± 25	-3.99 ± 3.1	1176 ± 14	-4.78 ± 1.69
	13460		1179 ± 16	-5.11 ± 2.01		

Sample type	*Sample number (GrM)*	*Calendar age (year CE)*	*^{14}C age (year BP)*	*Δ^{14}C (‰)*	*Averaged ^{14}C age (year BP)*	*Averaged Δ^{14}C (‰)*
C (Juniper)	16818	976	1136 ± 15	-23.32 ± 1.82	1136 ± 15	-23.32 ± 1.82
	16819	978	1108 ± 15	-20.15 ± 1.82	1108 ± 15	-20.15 ± 1.82
Sierra Nevada (USA)	16820	980	1113 ± 18	-20.99 ± 2.19	1113 ± 18	-20.99 ± 2.19
	16821	982	1143 ± 16	-24.88 ± 1.94	1143 ± 16	-24.88 ± 1.94
	17189	984	1120 ± 16	-22.32 ± 1.94	1120 ± 16	-22.32 ± 1.94
	17190	986	1101 ± 16	-20.24 ± 1.95	1102 ± 11	-20.36 ± 1.34
	17201		1102 ± 16	-20.36 ± 1.95		
	17191	988	1116 ± 18	-22.31 ± 2.19	1116 ± 18	-22.31 ± 2.19
	17192	989-991	1105 ± 19	-21.20 ± 2.31	1096 ± 12	-20.11 ± 1.46
	17202		1089 ± 16	-19.25 ± 1.95		
	17193	993	1047 ± 16	-14.47 ± 1.96	1047 ± 16	-14.47 ± 1.96
	17195	995	1035 ± 16	-13.23 ± 1.96	1035 ± 16	-13.23 ± 1.96
	17196	997	1045 ± 16	-14.70 ± 1.96	1045 ± 16	-14.70 ± 1.96

Table S1a, S1b, S1c, S1d. Raw radiocarbon measurements, expressed as ^{14}C age and Δ^{14}C

S2 – RAW DATA (PREVIOUSLY PUBLISHED)

**Event-775 (Miyake et al., 2013)**		
Calendar age (year CE)	*Δ^{14}C (‰)*	*± (‰)*
730	-11.8	2.1
732	-14.5	2.1
734	-10.4	2.1
736	-10.6	2.6
738	-13.2	2.1

740	-12.8	2.1
742	-17.9	2.1
744	-13.6	2.2
746	-15.3	2.1
748	-15.9	2.6
750	-16.0	1.8
752	-17.5	2.0
754	-20.4	2.0
756	-20.9	2.0
758	-18.0	2.0
760	-17.9	2.0

Event-994 (Miyake et al., 2013)		
Calendar age (year CE)	***$\Delta^{14}C$ (‰)***	***± (‰)***
988	-21.9	1.8
989	-20.2	1.9
989	-21.3	1.8
990	-25.3	2.9
990	-22.5	1.7
991	-21.5	1.5
991	-23.1	1.9
992	-22.8	2.0
992	-24.4	1.9
993	-20.7	1.6
993	-25.3	1.7
994	-11.5	2.0
994	-14.0	1.8
995	-12.9	1.5
995	-18.1	1.7
996	-11.3	2.0
996	-16.7	1.7
997	-14.2	2.0
997	-16.4	1.7
998	-15.3	2.0
999	-13.3	1.6
1001	-15.0	2.9
1003	-17.0	2.6
1005	-13.8	2.6
1007	-21.1	2.6
1009	-20.1	2.6
1011	-23.0	2.6
1013	-20.5	2.6
1015	-17.9	2.6
1017	-20.8	2.1
1019	-22.0	2.1

1021	-19.2	3.6

Event-994 (Menjo et al., 2005)		
Calendar age (year CE)	***$\Delta^{14}C$ (‰)***	***± (‰)***
996	-14.35	2.55
998	-16.59	2.55
1000	-15.90	2.55
1001	-15.33	2.55
1002	-15.35	2.55
1003	-18.36	2.55
1004	-16.78	2.55
1005	-19.28	2.55
1006	-18.96	2.55
1007	-18.64	2.55
1008	-20.42	1.90
1009	-20.47	1.88
1010	-16.98	1.99
1011	-19.69	1.98
1012	-16.50	1.98
1013	-15.27	1.99
1014	-18.54	2.82
1015	-12.13	2.82
1016	-16.98	2.82
1017	-14.19	2.82
1018	-19.03	2.55
1019	-14.35	3.20
1020	-22.97	3.20
1022	-20.79	3.20
1024	-14.28	2.14

Event-994 (Damon et al., 1995)		
Calendar age (year CE)	***$\Delta^{14}C$ (‰)***	***± (‰)***
1004	-14.6	3.9
1005	-18.7	4.0
1006	-21.8	4.0
1007	-19.3	3.7
1008	-21.1	3.4
1009	-22.2	3.8
1010	-15.2	3.3
1011	-10.00	3.5
1012	-17.1	3.3
1013	-11.1	3.2

1014	-16.7	3.0
1015	-11.8	4.1
1016	-18.3	3.4

**Event-994 (Dee et al., 2016)**		
Calendar age (year CE)	***$\Delta^{14}C$ (‰)***	***± (‰)***
1004	-13.20	3.45
1005	-23.59	3.41
1006	-12.46	3.20
1007	-17.48	3.80
1008	-23.58	3.41
1009	-24.55	3.53
1010	-16.25	3.80

Table S2a, S2b, S2c, S2d, S2e. Previously published ^{14}C measurements, expressed in $\Delta^{14}C$, utilized for the study of Event-775 and Event-994.

S3 – DUPLICATES AND CHI SQUARED TEST

Calendar year (year CE)	*Sample name*	*^{14}C age (year BP)*	*Uncertainty (year BP)*	*T-Statistic*	*Chi-Square Test*
Sample A					
1853	GrM-13331	124	15	0.024	**Pass**
	GrM-13404	129	16	0.028	
	**Weighted Average**	**126**	**11**	**0.052**	
1856	GrM-13334	169	16	1.598	**Pass**
	GrM-16797	131	15	1.404	
	**Weighted Average**	**149**	**11**	**3.002**	
1859	GrM-12716	142	13	0.022	**Pass**
	GrM-16798	153	14	0.421	
	GrM-18107	138	13	0.207	
	**Weighted Average**	**147**	**10**	**0.650**	
1860	GrM-12717	114	13	1.423	**Pass**
	GrM-13335	153	16	2.156	
	**Weighted Average**	**130**	**10**	**3.579**	
1862	GrM-12721	127	11	0.243	**Pass**
	GrM-12727	140	13	0.340	
	**Weighted Average**	**132**	**8**	**0.583**	
1864	GrM-13336	146	15	0.140	**Pass**
	GrM-13405	134	16	0.159	
	**Weighted Average**	**140**	**11**	**0.299**	
1865	GrM-12725	139	11	0.023	**Pass**
	GrM-12729	135	13	0.032	
	**Weighted Average**	**137**	**8**	**0.055**	
Sample B1					
774	GrM-16814	1261	16	3.486	**Fail**
	GrM-17197	1333	19	4.916	
	**Weighted Average**	**1291**	**12**	**8.402**	

777	GrM-16816	1193	16	1.000	Pass
	GrM-17198	1161	16	1.000	
	**Weighted Average**	**1177**	**11**	**2.000**	
**Sample B2**					
770	GrM-11849	1314	17.5	1.9	**Pass**
	GrM-12731	1286	14	0.1	
	GrM-12786	1276	16	0.8	
	GrM-14094	1290	11	0.0	
	**Weighted Average**	**1290**	**7**	**2.7**	
771	GrM-10567	1312	22.5	2.314	**Pass**
	GrM-11774	1267	15	0.516	
	GrM-12686	1250	37.5	0.548	
	**Weighted Average**	**1278**	**12**	**3.378**	
772	GrM-10568	1295	22.5	0.4	**Pass**
	GrM-12179	1286	14	0.1	
	GrM-12737	1266	14	1.2	
	GrM-12739	1271	14	0.6	
	GrM-14093	1292	11	0.9	
	**Weighted Average**	**1282**	**6**	**3.2**	
773	GrM-10569	1315	22.5	0.107	**Pass**
	GrM-13095	1312	19	0.053	
	GrM-13096	1298	19	0.257	
	**Weighted Average**	**1308**	**12**	**0.417**	
775	GrM-10572	1205	25	0.064	**Pass**
	GrM-17405	1215	19	0.037	
	**Weighted Average**	**1211**	**15**	**0.101**	
776	GrM-10574	1125	25	0.469	**Pass**
	GrM-13098	1152	19	0.271	
	**Weighted Average**	**1142**	**15**	**0.739**	
777	GrM-10575	1165	25	0.233	**Pass**
	GrM-14839	1148	16	0.095	
	**Weighted Average**	**1153**	**13**	**0.328**	
778	GrM-10577	1170	25	0.005	**Pass**
	GrM-14440	1173	15	0.102	
	GrM-15240	1162	16	0.150	
	**Weighted Average**	**1168**	**10**	**0.258**	
779	GrM-10578	1170	25	0.065	**Pass**
	GrM-13460	1179	16	0.027	
	**Weighted Average**	**1176**	**13**	**0.092**	
**Sample C**					
986	GrM-17190	1101	16	0.001	**Pass**
	GrM-17201	1102	16	0.001	
	**Weighted Average**	**1102**	**11**	**0.002**	
989-991	GrM-17192	1105	19	0.243	**Pass**
	GrM-17202	1089	16	0.172	
	**Weighted Average**	**1096**	**12**	**0.415**	

Table S3a. Details about averaging of duplicate measurements: T-statistics and Chi-Squared test

S4 – ADDITIONAL FIGURES

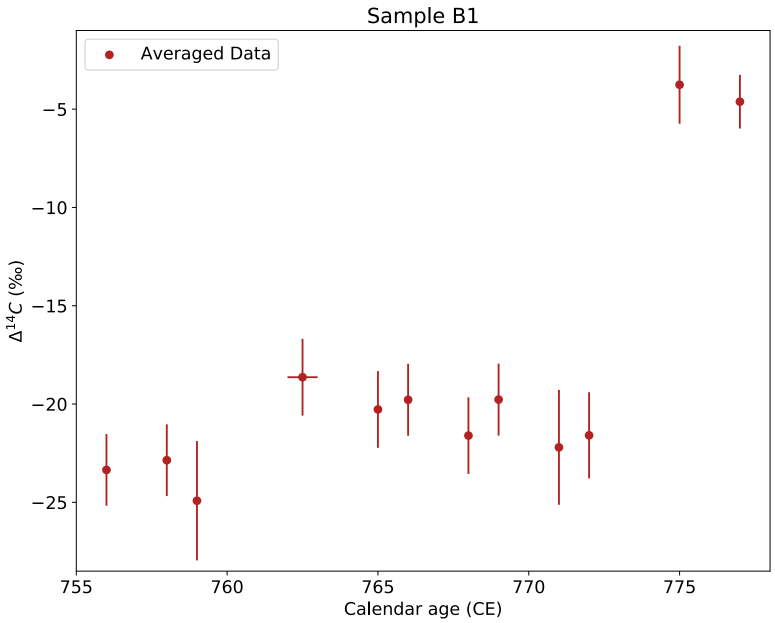

Figure S4a. **Results of the radiocarbon measurements over sample B1, concerning Event-775. Multiple measurements over the same calendar year have been averaged (see S3), and data points are shown with 1-sigma error bars.**

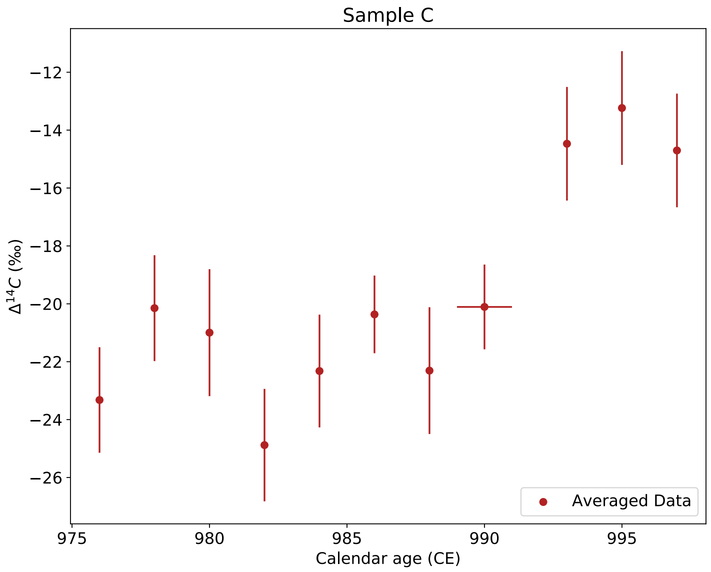

Figure S4b. **Results of the radiocarbon measurements over sample C, concerning Event-994. Multiple measurements over the same calendar year have been averaged (see S3), and data points are shown with 1-sigma error bars.**

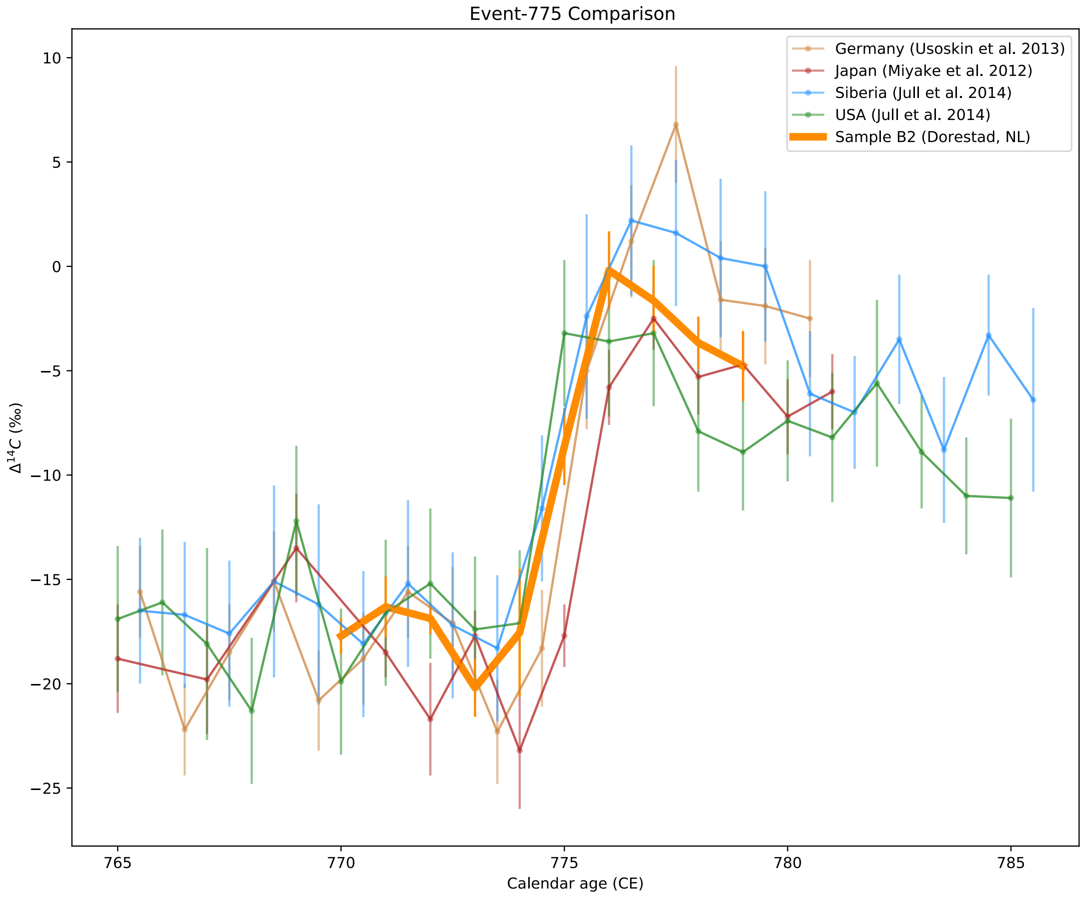

***Figure S4c. In orange,* results of the radiocarbon measurements over sample B2, concerning Event-775. Multiple measurements over the same calendar year have been averaged (see S3), and data points are shown with 1-sigma error bars. In other colors, radiocarbon measurements over the same event, previously published in other studies. Our measurements (in orange) sit perfectly in between the measurements of earlier analysis of other laboratories.**

S5 – INFO ABOUT SUNSPOT RECORD

All the data used for the study of the Sunspot Record in conjunction with the Carrington Event, were downloaded, as freely available, from the website of SILSO (Sunspot Index and Long-term Solar Observation) (http://www.sidc.be/silso/). SILSO is part of the Solar Influences Data Analysis Center (SIDC), which in turn is part of the Royal Observatory of Belgium and a partner in the Solar Terrestrial Center of Excellence (STCE). The data downloaded and used is the 'daily total sunspot number'.

S6 – DENDROCHRONOLOGY

Sample A

Wood: Oak
Site: Mapledurham, England
Dendro-code: MAP71x
Laboratory: Oxford Dendrochronology Laboratory,
Daniel Miles, daniel.miles@arch.ox.ac.uk

Sample B1

Wood: Juniper
Site: Sierra Nevada Mountains, California
Dendro-code: CS9d1
Laboratory: Oxford Dendrochronology Laboratory,
Daniel Miles, daniel.miles@arch.ox.ac.uk

Sample B2

Wood: Oak
Site: Dorestad Haven, The Netherlands
Project ID: P2016003
Arch. number: 357-8-38
Dendro-code: D1600030
Laboratory: Netherlands Cultural Heritage Agency (Rijksdienst voor het Cultureel Erfgoed),
Esther Jansma, E.Jansma@cultureelerfgoed.nl
Petra Doeve, P.Doeve@cultureelerfgoed.nl

Sample C

Wood: Juniper
Site: Sierra Nevada Mountains, California
Dendro-code: CS9d2
Laboratory: Oxford Dendrochronology Laboratory,
Daniel Miles, daniel.miles@arch.ox.ac.uk

S7 – CUBIC SPLINE INTERPOLATION AND RESIDUALS

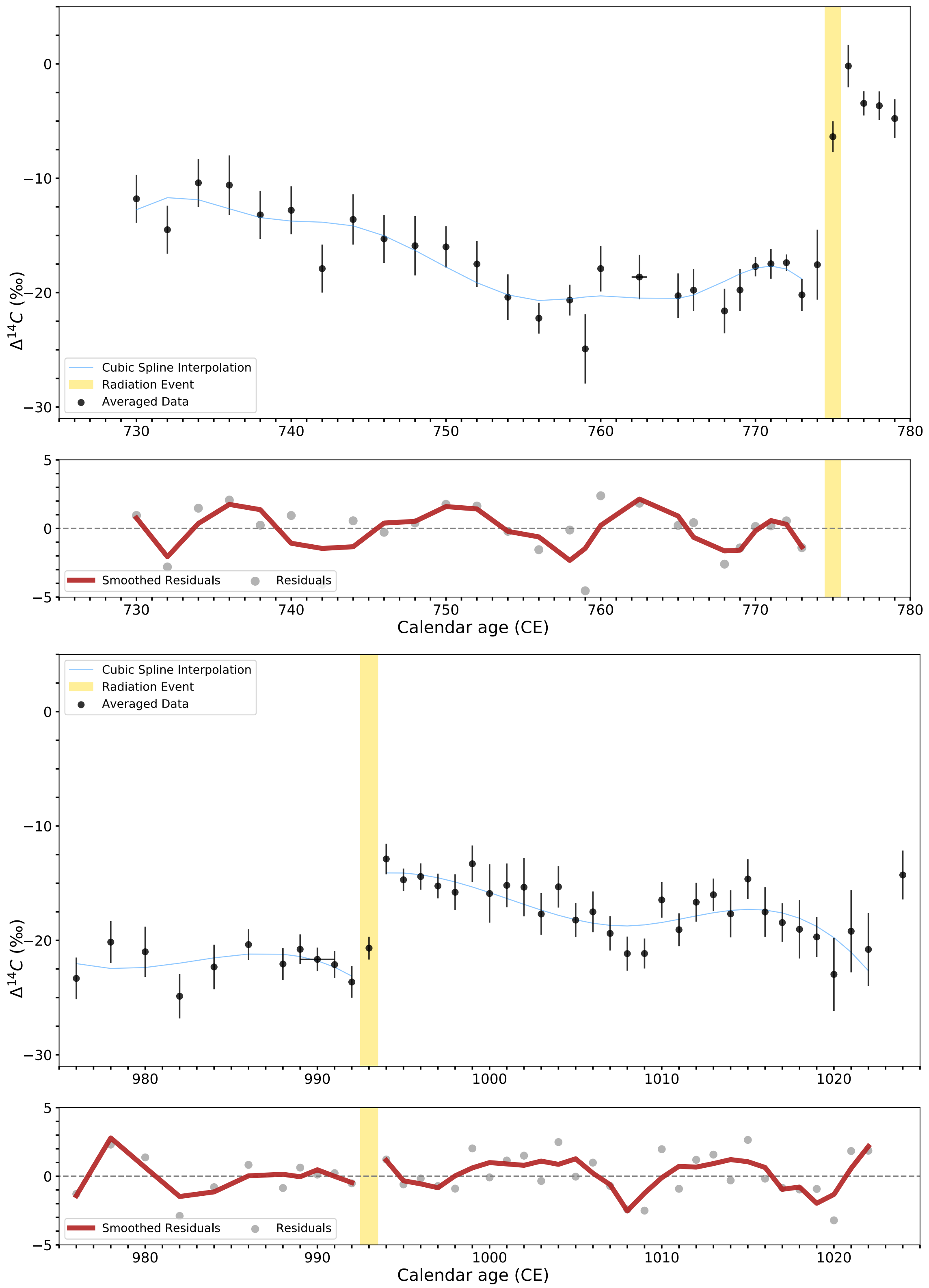

Figure S7a. Results of the analysis over the Event-775 (above) and Event-994 (below). In the top row, averaged data points with 1-sigma error bars (black) and time location of the radiation event (yellow). In the bottom row, residuals of the spline fit (gray dots) and smoothed residuals obtained through a Savitzky-Golay filter (red line).

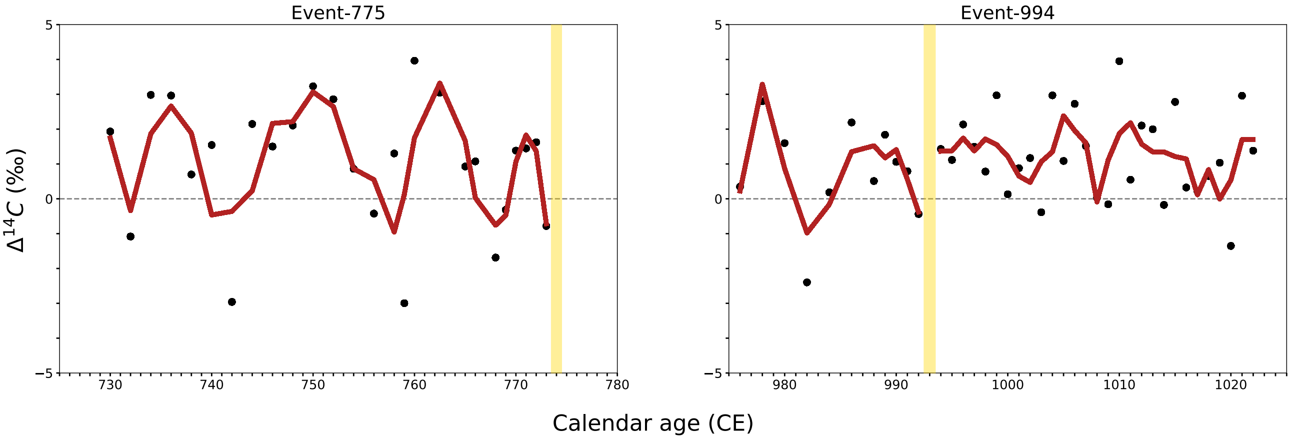

Figure S7b. Residuals of the cubic spline interpolation (gray dots) and smoothed residuals (red line) over 1,000 times Monte Carlo resampling of the normally distributed experimental data analyzed in Figure 3 and 4. The results over Event-775 is in accordance with the result of the band-pass filter over the same event. The result over Event-994 also coincides with the result of the band-pass filter over the same event, but only in the period before the spike. After the spike, the data is affected by the radiation event and the random resampling disrupts any periodicity.
